# The interconnected and cross-border nature of risks posed by infectious diseases

**DOI:** 10.3402/gha.v7.25287

**Published:** 2014-10-10

**Authors:** Jonathan E. Suk, Thomas Van Cangh, Julien Beauté, Cornelius Bartels, Svetla Tsolova, Anastasia Pharris, Massimo Ciotti, Jan C. Semenza

**Affiliations:** 1 European Centre for Disease Prevention and Control, Stockholm, Sweden; 2 Global Public Health Unit, School of Social and Political Science, University of Edinburgh, Edinburgh, UK

**Keywords:** globalization, climate change, epidemic, infectious disease, health determinants, Ebola

## Abstract

Infectious diseases can constitute public health emergencies of international concern when a pathogen arises, acquires new characteristics, or is deliberately released, leading to the potential for loss of human lives as well as societal disruption. A wide range of risk drivers are now known to lead to and/or exacerbate the emergence and spread of infectious disease, including global trade and travel, the overuse of antibiotics, intensive agriculture, climate change, high population densities, and inadequate infrastructures, such as water treatment facilities. Where multiple risk drivers interact, the potential impact of a disease outbreak is amplified. The varying temporal and geographic frequency with which infectious disease events occur adds yet another layer of complexity to the issue. Mitigating the emergence and spread of infectious disease necessitates mapping and prioritising the interdependencies between public health and other sectors. Conversely, during an international public health emergency, significant disruption occurs not only to healthcare systems but also to a potentially wide range of sectors, including trade, tourism, energy, civil protection, transport, agriculture, and so on. At the same time, dealing with a disease outbreak may require a range of critical sectors for support. There is a need to move beyond narrow models of risk to better account for the interdependencies between health and other sectors so as to be able to better mitigate and respond to the risks posed by emerging infectious disease.

The Ebola outbreak of 2014 has demonstrated very clearly the vast harm that infectious diseases can cause when combined with other exacerbating factors. Poor health systems, a slow international response, low public trust in government and medicine in the outbreak area, the introduction of the virus to urban settings, and social practices such as burial rites that increase the risk of contagion have created a ‘perfect storm’ for Ebola transmission (1). The gravity of the Ebola outbreak is demonstrated by thousands of deaths,^1^ and by the potential for further spread. The interconnected and cross-border nature of the threat has facilitated Ebola transmission in West Africa, and it has also led to cases appearing in both the United States and continental Europe.

As the global community mobilises in response to Ebola, it is clear that there is a need to better acknowledge that the spread of infectious diseases is related to the circumstances in which human society is organised. This has long been the case; human interactions with animal hosts have led to infectious disease outbreaks dating at least as far back as the Justinian Plague (541–542 AD), while global trade and travel have facilitated disease transmission, from the plague in the 14th century, smallpox in the 16th century, to SARS or a novel influenza virus in the 21st century (2–4). Indeed, it is often noted that in today's interconnected world, a disease can spread nearly anywhere on Earth within 24 hours. But infectious disease risks are not simply a by-product of globalisation. As Ebola demonstrates, a great many factors, some not restricted to the disease control or public health arena, can influence the spread of infectious disease. Assessing disease-related health risks thus necessitates understanding where they might arise and how successful it might be in transmitting. The largest risks tend to occur when novel diseases appear, when familiar diseases appear in novel geographic locations, or when preventative disease control measures break down, whether due to socioeconomic inequalities, lack of resources, accident, or conflict.

Despite this, the interconnections and interdependencies that create (and are created by) emerging infectious diseases tend to be downplayed, to the detriment of public health. In order to address the topic, this paper provides disparate examples of infectious disease outbreaks, with the common theme being the many factors that combined to worsen the situation. ‘Infectious disease drivers and interconnected risk’ section will discuss the types of factors that can influence the spread of infectious disease. ‘Examples of interconnections and interdependencies in recent infectious disease events’ section will provide some examples of recent infectious disease incidents that have been created by the interconnectedness of factors introduced in the ‘Infectious disease drivers and interconnected risk’ section. These examples are primarily focused on the European Union (EU), the area in which the authors are based, but the global linkages have been emphasised. ‘Analysing interconnected and interdependent infectious disease risks’ section will discuss some of the integrated risk modelling approaches that have been developed in an attempt to monitor and even predict infectious disease transmission, and the ‘Conclusion’ section will summarise this paper by reiterating the need for and importance of recognising the interconnected and interdependent nature of infectious disease risks.

## Infectious disease drivers and interconnected risk

The concept of interconnected risks refers to the idea that seemingly disconnected risks are intrinsically linked to one another (5). In health, the concept of interconnected risk helps draw attention to the fact that factors traditionally viewed of as ‘external’ to health can nonetheless drive health risks. Emerging infectious diseases, for example, can easily be viewed as interconnected because they are affected by a wide range of ecological, political, and socioeconomic ‘drivers’ (6–8). They can also be viewed as interdependent because of their transboundary nature, and because risks from other sectors can interact synergistically or antagonistically. In short, the emergence of an infectious disease risk can amplify and be amplified by risks from other sectors. These could include employee absenteeism and workforce shortages, potential social discord, or severe disruptions in energy, healthcare delivery, transportation, or other critical sectors (9, 10).

There are many different ways in which the myriad factors that influence infectious disease could be categorised. For the sake of simplicity, the remainder of this discussion is derived from a study which grouped them according to three broad (and somewhat simplistic) categories: globalisation and environmental change; social and demographic factors; and health and food production (7). It should be noted that although in the following sections each set of factors will be discussed individually, they are themselves often interconnected, adding an additional layer of complexity to the issue. Furthermore, the examples provided below are merely indicative and not exhaustive.

### Globalisation and environmental change

A wide range of infectious disease drivers can be grouped under this category, including climate change, land-use patterns, global trade and travel, migration, and so on. Climate change involves mean temperature increases in many parts of the world, as well as increased likelihood of adverse or even extreme weather events (11–13). Many infectious diseases are temperature sensitive as many vectors and pathogens are dependent upon permissive ambient conditions. There is thus a substantial body of research that collectively demonstrates that warming will increase the transmission of vector-borne diseases in the geographic ranges of their distribution (14–18). Changing temperature and precipitation patterns can affect the habitats and population growth of cold-blooded disease vectors, such as mosquitoes and ticks, as well as the replication rates of infectious diseases within their hosts, and even the rates at which disease-carrying vectors bite humans (18–20).

Among the best substantiated indicators of the observed effects of climate change on infectious disease is evidence of an altitudinal increase of malaria in the highlands of Columbia and Ethiopia (21) and of the northerly expansion of the disease-transmitting tick species, *Ixodes ricinus*, in Sweden (22). Many modelling studies project significant shifts in the transmission of vector-borne diseases such as malaria (23, 24), dengue (25), and Chikungunya (26) under climate change scenarios, but it is important to note that the extent of observed changes will depend on the presence or absence of mitigating measures, such as vector control or socioeconomic development (27, 28). Other examples of infectious diseases in Europe anticipated to be affected by climate change include West Nile virus (29), salmonella (30), campylobacter, and cryptosporidium (31, 32).

Land-use patterns, meanwhile, are a crucial driver of infectious disease emergence. It has been estimated that more than 60% of human pathogens are zoonotic (i.e. diseases of animals that can be transmitted to humans) (33). Many human land-use activities, including agriculture, irrigation, hunting, deforestation, and urban expansion, can cause or increase the risk of zoonotic and food- and water-borne diseases (33, 34). For example, one consequence of urban sprawl and deforestation is that wildlife may increasingly need to find new habitats in urban or abandoned environments, which could lead to increased human exposures to infectious pathogens. Meanwhile, the density of human population, also associated with increasing urbanisation, has also been shown to be linked to the emergence of many infectious diseases (35).

Intensified global trade and travel, not to mention migration, render political borders irrelevant and create further possibilities for global disease transmission (36–38). There are numerous examples of the arrival, establishment, and spread of ‘exotic’ pathogens to new geographic locations, including malaria, dengue, Chikungunya, West Nile, and bluetongue in recent years, aided by shipping or other trade routes (36). This process is facilitated when the environmental conditions in different parts of the world share common characteristics (36). Meanwhile, numerous vaccine-preventable diseases, such as polio, meningitis or measles, can also be introduced or reintroduced to susceptible populations as a consequence of international travel (39).

### Social and demographic factors

Social and demographic contexts can significantly influence the transmission of infectious disease, while also creating increased vulnerabilities for some population subgroups. The elderly are at greater risk of many infectious diseases, and the ageing trend in many high-income countries could increase the challenges related to nosocomial (hospital-acquired) and nursing-home acquired infections. An additional challenge related to population ageing is that the share of employed workers in a country decreases. The combination of more people to care for and fewer tax-related revenues may challenge publicly financed public health and disease control programmes (7).

When persons from regions with high endemicity of a given disease move to ones with lower endemicity, new challenges for public health are created. In addition migrant communities can be highly vulnerable to certain infectious diseases. In the EU, for example, approximately 37% of HIV cases reported in 2011 were among people born abroad, and the equivalent number of cases for tuberculosis was 25% (40). Similarly, migrants suffer from a higher burden of chronic hepatitis B infections (41).

It is widely established that socially and economically disadvantaged groups suffer disproportionally from disease (42). This is applicable to infectious disease burdens in both high- and low-income settings (43, 44). Income inequalities are generally widening globally, and this appears to be have been exacerbated in many countries due to the global economic crisis (45). Rising unemployment and the prospect of public health budget cuts can increase the risk of infectious disease transmission (44, 46), with a prominent example being an outbreak of HIV among people who inject drugs (PWID) in Greece (see ‘Measles among Roma in Bulgaria and HIV among PWID in Greece: the impact of socioeconomic contexts’ section) (47, 48). In a similar fashion, it has been speculated that tuberculosis rates could rise in some countries in Central and Eastern Europe (49).

Social trends and behaviours can also play a significant role in infectious disease transmission. The most notable example would be vaccine hesitancy, the phenomenon through which vaccination coverage rates remain suboptimal due to the varying and complicated reasons that individuals may have for not getting vaccinated (50, 51). In some cases, this might be related to misconceptions about the safety or efficacy of vaccines (50, 52), whereas in others this may be related to religious or cultural beliefs (53).

### Health and food production

The financing, provision, and quality of healthcare systems; the availability of vaccines, antivirals, and antibiotics medicines, and appropriate compliance to treatment protocols are all important determinants of infectious disease transmission. Although the correlation between healthcare system financing and efficacy is not perfect, recent budget cuts to healthcare are an important consideration when anticipating infectious disease risk. In part related to the global economic crisis, it has been reported that many high-income governments have introduced policies to lower spending through cutting the prices of medical products and, for example, through budget restrictions and wage cuts in hospitals (54). There are many indirect and direct pathways through which budget cuts could affect disease transmission; to provide just one example, it has been estimated that 20–30% of healthcare-associated infections are preventable with intensive hygiene and control programmes^2^ – should investments in this area diminish, then healthcare-acquired infections could become an even more problematic issue. There are currently roughly 4.1 million healthcare-associated infections each year in the EU alone.^3^

A broader issue related to healthcare provision is population mobility for both healthcare professionals and patients who might increasingly seek work or healthcare in other countries – the provision of cross-border healthcare and the mitigation of cross-border health threats will necessitate collaboration across borders (55, 56) and solutions for the brain-drain of medical personnel from resource-poor countries (57). Also related to the healthcare provision and practice is the over-prescription or overuse of antibiotics. In combination with a lag in pharmaceutical innovation, rapid transmission, and poor infection control measures, this has driven resistance of organisms such as methicillin-resistant *Staphylococcus aureus*, or extended-spectrum beta-lactamases, and carbapenemase-producing gram-negatives such as *Klebsiella pneumoniae carbapenemase* (KPC) (58). Antimicrobial resistance is currently one of the major health risks facing society (59).

Food production systems remain a persistent source for human infectious diseases. Attempts are underway to estimate the global burden of food-borne disease (60), which is likely substantial. Many factors in food production affect human health. A vast range of familiar human pathogens can be acquired through the consumption of animal products and other disease drivers, such as global travel, further provoke this (61). In addition to farmed animals, the hunting and slaughtering of wild animals has led to the emergence of more exotic pathogens: SARS originated in wildlife markets and restaurants in southern China (62) and HIV and Ebola have both been linked to the hunting or slaughtering of primates and other wild animals (33, 63, 64). The density and health of livestock, meanwhile, have been linked to disease in humans (65, 66). Although inconclusive, there is some evidence to suggest that livestock production may lead to increased antibiotic resistance in human pathogens. There are certainly many pathways by which drug resistant pathogens could transmit from livestock to humans, including environmental contamination by excreted veterinary antibiotics (33, 67, 68).

## Examples of interconnections and interdependencies in recent infectious disease events

It is noteworthy that many of the disease drivers discussed above are, from the perspective of other sectors, risks in their own rights. The World Economic Forum's Global Risks Report 2013 identified two infectious disease-specific risks, vulnerability to pandemics and antibiotic-resistant bacteria (highlighting the latter) (69). Even more noteworthy, *apropos* the discussion from ‘Infectious disease drivers and interconnected risk’ section, many of the risks identified in that report are themselves exacerbating factors for the spread of infectious disease. These include: major systemic financial failure; failure of climate change adaptation; severe income disparity; mismanagement of population ageing; terrorism; land and waterway use mismanagement; mismanaged urbanisation; and species overexploitation (69).

It is instructive to provide a few examples of how such risks are interconnected in the context of infectious disease. Literally, hundreds of examples from around the world could have been selected. One study identified 335 events leading to an emerging infectious disease between 1940 and 2004, identifying numerous infectious disease ‘hotspots’ across the globe – areas in which a disproportionately high number of disease emergencies have concentrated (35). Western Europe is identified as one such hotspot, and as the region with which the authors of this chapter are most familiar, the examples below will be slightly biased towards Europe. Nonetheless, while highlighting the interconnections between diverse disease drivers, the global interdependencies inherent to these examples will also be evident. The following examples have been selected to interrogate how different combinations of drivers have affected recent infectious disease risks. In ‘Dengue and Chikungunya in Europe: links with globalisation and environmental change’ section, the links between environmental factors, climate change, and global trade and travel are discussed. In ‘Measles among Roma in Bulgaria and HIV among PWID in Greece: the impact of socioeconomic contexts’ section, socioeconomic contexts and the impact of the financial crisis are examined for their influence on disease outcomes. ‘Avian influenza: human–animal interfaces and global travel’ section examines avian influenza in the context of human–animal interfaces and global travel, and ‘The impact of conflicts and population displacement on attempts to eradicate polio’ section investigates the impact of terrorism, conflict, and population displacements on infectious disease risk.

### Dengue and Chikungunya in Europe: links with globalisation and environmental change

The sustained transmission of a vector-borne disease requires the presence of a pathogen, a vector capable of transmitting that pathogen, and a susceptible human population (36). The interconnections between multiple disease drivers and interdependencies created by a globalised world have enabled the global expansion of vector-borne diseases, of which recent examples include the arboviruses dengue (70) and Chikungunya (71, 72).

One critical factor in the expansion of these diseases has been global trade. The mosquito species *Aedes albopictus*, also known as the Asian Tiger mosquito, is a secondary vector of dengue and Chikungunya and is thought to be the most invasive mosquito species of public health importance in the world (73). Over the past few decades, it has expanded from its original habitat in Asia to all populated continents in the world (74). It has been documented that this expansion has been enabled by global trade in used tires (73, 75).

In Europe, the climatic conditions have been permissive enough to enable *Ae. albopictus* to gradually expand (often via transportation networks) from its introduction in Italy, where it arrived in 1990 (76). Today, *Ae. albopictus* is established in many regions of the Mediterranean Basin, including in Spain, France, Italy, Croatia, and Greece. In addition, the mosquito has been introduced to regions as far north as Germany, the Czech Republic, and Slovakia.^4^ Models based on the known climatic determinants of *Ae. albopictus* suggest that many more areas of Europe could be suitable habitats for the mosquito (77) as well as for Chikungunya transmission, with some regions currently also amenable to dengue transmission (74). Under climate change scenarios, additional areas in Central and Western Europe, but fewer areas in southern Europe, could be climatically suitable (26, 78).

In a deeply interconnected world, there are numerous opportunities for viruses to be introduced into non-endemic regions (38). Each year, millions of people fly between regions of the world endemic to dengue or Chikungunya and European regions that are now home to *Ae. Albopictus*, whose ability to transmit Chikungunya increased after a single genetic mutation in the Chikungunya virus (79). In 2007, a traveller from India, infected with Chikungunya, was bitten by mosquitoes in Italy, leading to a local outbreak in which over 200 people were infected (80). Dengue, meanwhile, which is now endemic in many tropical and sub-tropical parts of the world (81), has also been transmitted by *Ae. albopictus* in Europe, as has been documented in France and Croatia (82, 83).

As a result of the continuing expansion of *Ae. albopictus* in Europe, aided by trade and travel networks, climatic conditions, and genetic evolution, two diseases previously ‘exotic’ to Europe now pose a persistent infectious disease risk. This sort of risk is clearly not restricted to Europe; in late 2013, Chikungunya was introduced and then initially locally transmitted in a few Caribbean islands, creating a high risk of further disease spread across the region.^5^ By early September, 2014, Chikungunya transmission has been reported in 31 countries and territories in the Caribbean and the Americas, with over 700,000 suspected cases and over 8,600 confirmed autochthonous cases.^6^

### Measles among Roma in Bulgaria and HIV among PWID in Greece: the impact of socioeconomic contexts

Socioeconomic contexts affect the spread of disease. When financial circumstances deteriorate, the most vulnerable members of society are at even greater risk of infectious disease. Two examples in this section demonstrate the links between public health provision, income disparities, and infectious disease.

Economic progress of the Roma population in Bulgaria has experienced a number of setbacks, ever since the political and economic transition of the late 80s (84, 85). Discrimination, poor educational attainment, lack of occupational training, poor access to labour markets, and geographic isolation have all contributed to high unemployment rates (86). The lack of job opportunities discourages further training and education, thereby perpetuating a vicious circle of poor education, poor working opportunities, and worsening living conditions (85). For example, Roma are five times more likely to feel their health is threatened due to the unhygienic conditions in which they live than is the Bulgarian majority population (87). These circumstances, connected to broader cultural, demographic, and economic risks, have precipitated one of the worst measles outbreaks in Europe of the past decades. Over 24,000 individuals fell ill and 24 died over a time period of a few months, starting in April 2009 (88). Approximately 90% of the cases were from the Bulgarian Roma community despite the fact that only 4% of the general Bulgarian population are Roma. In this outbreak, the risk for developing severe medical complications such as pneumonia or encephalitis from a measles infection was linked to lower maternal education levels, lower child immunisation coverage, and the absence of household incomes (86). Despite Bulgarian children being eligible for free basic medical services, almost 95% of cases had not received the full course of measles–mumps–rubella (MMR) vaccination.

Although the root causes of measles outbreak are multifactorial, it is noteworthy that Bulgaria had one of the lowest rates of total governmental spending over gross domestic product in 2009 of the EU (37% vs. 49%, respectively) (89). Roma communities remain susceptible to communicable diseases such as the measles outbreak in 2009; outbreaks of hepatitis A, tuberculosis, and poliomyelitis have also disproportionally affected the Roma ethnic group (90, 91).

Another highly vulnerable group for infectious diseases in Europe are PWID. In the wake of the global economic crisis, many health professionals across Europe anticipated adverse effects in relation to infectious disease incidence and control (92). An observed rapid increase in reported HIV cases among PWID in both Greece and Romania was thought to be linked to the economic crisis (48). In Greece, for example, HIV incidence among PWID increased by 1600% in 2011 (47). From an epidemiologic standpoint, it is difficult to causally link the economic crisis with an upsurge in HIV in a particular subpopulation. Nonetheless, there are potential causal factors worth noticing. Yearly change in the Greek GDP has been found to be inversely associated with HIV case reports, homelessness, unemployment among PWID, and HIV prevalence among drug injectors seeking drug treatment in Athens (47). Meanwhile, causal pathways have been hypothesised to include the following factors: the economic recession increased income disparities, leading to increased homelessness; this contributed to increased injecting network sizes among PWID, which were then exposed to new introductions of HIV from migrant communities, themselves subject to difficult socioeconomic circumstances in their home countries (47). Transmission risk was intensified by low levels of available injecting equipment and other prevention services (47). Although difficult to demonstrate the causal linkages, this scenario suggests that the economic crisis, which originated in the financial sector, may have contributed to a large upswing of HIV in a vulnerable population, making conditions among PWID even more tenuous and, simultaneously, leading to fewer resources dedicated to harm prevention.

### Avian influenza: human–animal interfaces and global travel

Specific subtypes of influenza A viruses that circulate in birds can infect humans. Influenza infections of avian origin occur through both direct and indirect exposure to infected animals, whether alive or dead. One important unique feature of influenza viruses is their ability to cause both annual epidemics (seasonal influenza) and, from time to time, more serious pandemics (93). Each emerging strain has the potential to become seasonal.

Birds are a natural reservoir for influenza viruses, and A virus subtypes H5, H7 and H9 have all led to outbreaks in human populations (94). In recent years, the most significant outbreaks have been related to H5N1 and H7N9. Although a limited number of human cases infected with influenza A(H5N1) has been reported, the high case fatality and its potential ability to adapt to human hosts have raised concern at the global level (95). More recently, in the spring of 2013, 145 people in China were infected by the avian influenza strain A(H7N9), leading to 45 deaths (96). The virus was detected in poultry but also in the environment. The closure of live poultry markets in April 2013 did lead to a dramatic drop in the number of cases (97) although sporadic cases have been reported through the end of 2013.^7^

A broad combination of factors can trigger and sometimes amplify avian influenza outbreaks (98, 99). Ecological and environmental factors play a key role. Population density of both human and animals – as well as the proximity between them – are known risk factors for avian influenza infections in humans. Live bird markets and human consumption patterns of poultry and other avian species are also known to contribute to the risk of both influenza emergence as well as infection (100). Seasonality is another influencing factor, although different hypotheses exist as to why winter seasons are traditionally driving influenza transmission (101). Bird migratory patterns, particularly where migratory birds might interact with livestock poultry, create potential pathways for introduction of the virus into new regions. Air travel can quickly lead to the rapid global spread of influenza (3). Meanwhile, the level of available public health measures, from molecular surveillance to rapid vaccine production, are important determinants of the impact that any given influenza outbreak might have (102). Significantly, different avian influenza strains have different characteristics, further challenging the public health response. For example, influenza A(H5N1) is highly pathogenic in birds, leading to natural sentinel surveillance systems, while influenza A(H7N) may circulate among healthy birds, thereby remaining undetected (103).

The global and sectoral interdependencies related to influenza have been well documented. A widespread pandemic could quickly disrupt activities in many sectors, including trade (including potential trade bans) (104), transportation, healthcare delivery, critical infrastructure, and so on. School closures and workplace absenteeism are oft-cited control measures that might also have substantial impact on society. Numerous economic estimates indicate that the direct costs (use of healthcare services) and indirect costs (productivity losses) related to seasonal influenza can amount to billions of dollars globally (105). An economic scenario analysis of pandemic influenza in the United Kingdom indicated potential costs of 0.5–1% of GDP in low fatality scenario and 3.3–4.3% in a high fatality scenario (106).

### The impact of conflicts and population displacement on attempts to eradicate polio

Political unrest, armed conflict, and related population displacements are well known to be severe barriers to the provision of public health programmes (including immunisation programmes). Needless to say, conflicts also lead to significant vulnerabilities among populations, whilst population movements create further opportunities for disease spread.

Complex security situations have played a role in slowing down global efforts at polio eradication. In Pakistan, two workers associated with the World Health Organization (WHO) and its polio vaccination campaign were shot dead in July, 2012 (107), and it has since been reported that 31 people associated with polio campaigns were killed in the country between July 2012 and December 2013; reportedly, polio workers have been targeted for killing with terrorist intentions.^8^ Vaccination campaigns in Pakistan have been further hindered by public mistrust (107), a situation that likely further deteriorated after it was revealed that one doctor had run a fake vaccination campaign as part of a collaboration with the US Central Intelligence Agency efforts to find Osama bin Laden (108, 109). Pakistan had reported 85 polio cases in 2013, up from 55 in 2012.^9^ Assuming that roughly 95% of polio infections are asymptomatic and another 4.5% have mild or non-paralytic symptoms,^10^ this means that at least a few thousand additional people could be currently carrying and transmitting the virus in the country. Meanwhile, polio eradication programmes have pushed back their target completion dates, and it is has been reported that 350,000 children in Pakistan's Federally Administered Tribal Areas have not received polio vaccinations since mid-2012.^11^

Spill-over into neighbouring countries is one consequence of halted polio eradication. It appears that an introduction of polio from Pakistan into the broader region led to viral circulation in Israel, Egypt and, potentially, Syria (110). In Israel, circulation of the poliovirus occurred despite an estimated vaccination coverage of greater than 95% of the general population (110, 111). In Syria, 17 laboratory-confirmed cases were identified in 2013, implying widespread circulation of poliovirus (111).

The combination of conflict and circulating poliovirus is potentially highly problematic, as there are currently more than two million Syrian refugees globally, with an increase of 700,000 since July 2013 (111). This vast number, with high numbers of children, many of whom may be unvaccinated, creates the potential for further regional spread of poliovirus. In a globalised world, this risk is not localised. In the EU, 10% of new asylum applications between January 1 and August 2013 were from Syria, while the number of undocumented migrants from Syria has also increased (111). Such circumstances increase the risk of poliovirus reintroduction and transmission.

The risk of further global spread of wild poliovirus was recently acknowledged by WHO when it declared a Public Health Emergency of International Concern in May 2014.^12^ As of summer 2014, there are four countries exporting polio: Equatorial Guinea, Cameroon, Pakistan, and Syria. There are additionally six polio-affected countries: Afghanistan, Ethiopia, Iraq, Israel, Nigeria, and Somalia.^13^ As WHO noted:

The consequences of further international spread are particularly acute today given the large number of polio-free but conflict-torn and fragile States which have severely compromised routine immunization services and are at high risk of re-infection.^14^

Complicating the matter even further is the fact that alongside broader political issues that affect the possibility of implementing vaccination programmes, personal beliefs about the safety and benefits of vaccines are modulated by social ties, religious beliefs, and familial values. All of these factors affect vaccination uptake rates, as recent studies in both Pakistan and Nigeria have shown (112, 113).

There are many tragic dimensions to humanitarian crises and conflict-ridden regions, which aside from numerous other challenges must also deal with potentially explosive infectious disease outbreaks. This example demonstrates that inadequate global coordination of the health response (114), in combination with the interconnection of key risks such as low vaccination coverage (50, 115), conflict and terrorism, and population displacement, have the potential to amplify an already serious risk to global health.

## Analysing interconnected and interdependent infectious disease risks

There is a growing awareness within the health sector that the wide range of factors – many of them risks generated from other sectors – that can combine to affect the transmission of infectious disease need to be better and more holistically monitored, assessed, and acted upon. This is reflected by calls to approach the topic from a broader systems perspective (7, 33, 116–119), which tends to emphasise the need for integrating insights from multiple sectors and disciplines. Similarly, the One Health approach, which recognises the intimate relationship between environmental conditions, animal health, and human health, has been promoted at the global level^15^; ways of operationalising One Health concepts have also gained traction (120). Growing attention to the social determinants of health, meanwhile, is another crucial development for assessing and monitoring infectious-disease-related risks.

In concert with the growing awareness of the need to broadly assess the interconnected nature of infectious disease risk, there have been growing calls to develop tools and methodologies for predictive modelling of infectious disease outbreaks that assess, to some degree, the amplifying role that non-health factors play in propagating disease risk (17, 121, 122). Lagging behind have been analyses of vulnerabilities as well as strong evidence bases on the status and efficacy of public health preparedness (123) – two critical determinants of the magnitude of impact that any disease outbreaks might have.

There are a wide range of approaches to predictive infectious disease modelling (122). One of the more promising developments has been research into the links between environmental variables and infectious disease spread. As discussed in the ‘Infectious disease drivers and interconnected risk’ section, many environmental drivers can be considered as epidemic precursors of disease, and thus monitoring changes in environmental conditions can help anticipate or even forecast an upsurge of disease (121). In recent years, work in this area has been greatly enabled by rapid developments in Geographic Information Systems, which have facilitated the management and use of spatial data for analytic epidemiology. Climatic, weather, and environmental data can now be linked and integrated with data on health, disease vectors, and so on, to provide support tools for decision makers (121).

To illustrate how this has can be done, the European Centre for Disease Prevention and Control developed the European Environment and Epidemiology (E3) Network to help monitor environmental conditions related to infectious disease threats (121). The initial building-block of the E3 Network is data from the Emerging Diseases in a Changing European Environment project (EDEN), a research initiative funding by the Directorate-General for Research and Innovation of the European Commission.^16^ The aggregation of these data sets, and those continuously acquired from other sources, with regular outputs from advanced scientific analysis, serve as a starting point for analysis of disease risk and vulnerable regions.

This approach has been used to predict the environmental suitability of malaria transmission in Greece (124). Malaria was eradicated from Greece in 1974, but in 2009 (and subsequent years), locally acquired cases were identified. Remotely sensed data were used to describe the environmental and climatic conditions where future transmission could happen in Greece. Sea-level altitude and the mean and annual variation of land-surface temperature, both for daytime and night-time, were predictors in this model. Defining the areas of high risk helped guide the public health responses and to integrate preparedness and response activities, including targeted epidemiological and entomological surveillance, vector control activities, and awareness rising among the general population and health workers, in the areas environmentally suitable for transmission.

In another example, the E3 Network hosts a real-time model for the environmental suitability of *Vibrio spp*. in marine waters worldwide, taking into account temperature and salinity (125). It uses daily-updated remotely sensed data to assess environmental suitability conditions for *Vibrio spp*. infections according to a model developed by Baker-Austin et al. (126). Infections caused by Vibrio species (other than *V. cholerae*) can cause serious problems for immunocompromised persons, although the overall occurrence is generally low. Nonetheless, early information about the environmental suitability of *Vibrio spp*. infections will be of public health interest, and also demonstrates a proof-of-concept for predictive disease modelling more generally.

Linking data from other relevant fields is currently at a very early stage of development. Incorporating human behaviour into infectious disease modelling has thus far remained an understudied area (127). There are, however, recent promising approaches that involve tapping into mainstream and social media so as to measure and monitor issues such as vaccine hesitancy (128). The emerging field of digital epidemiology seeks to leverage the vast amount of digital information that exists and combine data relevant to both the transmission of disease as well as health behaviours (129). This field offers enormous promise but also needs to make progress in resolving questions surrounding methodologies, data quality and availability, and the privacy of online data.

## Conclusion

As has been stressed in this paper, infectious diseases are very often the product of a wide range of interacting and interconnected factors, and disease outbreaks create many cross-sectoral interdependencies. There is an emerging body of research on deploying a systems perspective of the many factors affecting infectious disease risks, and there are now even early stage predictive modelling frameworks that show promise. Information from these analyses can be used to set public health priorities and to inform civil society about potential consequences that factors external to health, such as global change, have on infectious disease control.

Moving forward, more research into the efficacy and status of public health preparedness and further work on the cross-sectoral dependencies created by disease outbreaks will be important areas for development. Meanwhile, methodological development of approaches for analysing priorities for public health preparedness activities and tools for facilitating cross-sectoral collaboration were recently highlighted by participants representing more than 30 countries at a Joint ECDC-WHO Consultation on Pandemic and All-Hazard Preparedness, held in November, 2013.^17^ These will be developed by ECDC in the coming years.

At the European policy level, the multifactorial, interconnected, and interdependent nature of infectious disease risks has been recognised. On 6 November 2013, a new EU Decision on serious cross-border threats to health entered into force.^18^ Two of the main features of this legal instrument are its ‘all-hazard’ approach and its emphasis on inter-sectoral collaboration. Both aspects are of strong relevance for an integrated approach towards interconnected risks.

The all-hazard component implies a shift from a cause-driven to an impact-driven assessment and management of health threats. In this sense, the Decision builds on the EU's extensive experience in the field of communicable diseases and expands it to other types of threats of biological, chemical, or environmental origin, ensuring a consistent European response across countries and across types of public health emergencies. The inter-sectoral component aims at enhancing the joint preparedness and response planning among European countries, resulting in comprehensive assessments of emergency situations and a formulation of responses addressing the whole range of their potential impacts. This Decision thus draws attention to the need for integrated approaches towards the identification, assessment and management of interconnected risks.

## Footnotes

1 http://apps.who.int/iris/bitstream/10665/135765/1/roadmapupdate3oct14_eng.pdf?ua=1, accessed October 7, 2014.

2 http://ecdc.europa.eu/en/healthtopics/Healthcare-associated_infections/Pages/index.aspx, accessed January 14, 2014.

3 http://ecdc.europa.eu/en/healthtopics/Healthcare-associated_infections/Pages/index.aspx, accessed January 14, 2014.

4 http://www.ecdc.europa.eu/en/healthtopics/vectors/vector-maps/Pages/VBORNET_maps.aspx, data as of October, 2013. Accessed January 12, 2014.

5 http://ecdc.europa.eu/en/press/news/_layouts/forms/News_DispForm.aspx?List=8db7286c-fe2d-476c-9133-18ff4cb1b568&ID=940&RootFolder=/en/press/news, accessed January 12, 2014.

6 http://www.paho.org/hq/index.php?option=com_topics&view=article&id=343&Itemid=40931, accessed September 13, 2014.

7 http://ecdc.europa.eu/en/press/news/_layouts/forms/News_DispForm.aspx?List=8db7286c%2Dfe2d%2D476c%2D9133%2D18ff4cb1b568&ID=930&RootFolder=%2Fen%2Fpress%2Fnews%2FLists%2FNews&Source=http%3A%2F%2Fecdc%2Eeuropa%2Eeu%2Fen%2Fhealthtopics%2Favian%5Finfluenza%2FPages%2Findex%2Easpx&Web=86661a14%2Dfb61%2D43e0%2D9663%2D0d514841605d, accessed January 10, 2014.

8 http://www.aljazeera.com/indepth/features/2013/12/pakistan-polio-workers-targeted-killing-201312118364851379.html, accessed January 14, 2014.

9 http://www.polioeradication.org/Dataandmonitoring/Poliothisweek.aspx, accessed January 14, 2014.

10 http://www.cdc.gov/vaccines/pubs/pinkbook/downloads/polio.pdf, accessed September 13, 2014.

11 http://www.cdc.gov/media/releases/2013/p1121-polio-progress.html, accessed January 14, 2014.

12 http://www.who.int/mediacentre/news/statements/2014/polio-20140505/en/, accessed July 16, 2014.

13 http://www.polioeradication.org/Infectedcountries/PolioEmergency.aspx, accessed July 16, 2014.

14 http://www.who.int/mediacentre/news/statements/2014/polio-20140505/en/, accessed July 16, 2014.

15 http://www.oie.int/en/for-the-media/editorials/detail/article/one-world-one-health/, accessed January 14, 2014.

16 http://www.eden-fp6project.net/, accessed January 14, 2014.

17 http://www.ecdc.europa.eu/en/publications/Publications/Joint-ECDC-WHO-Europe-Consultation-on-pandemic-and-all-hazard-preparedness-meeting-report.pdf, accessed July 14, 2014.

18 http://ec.europa.eu/health/preparedness_response/policy/decision/index_en.htm, accessed January 14, 2014.
