# Corrigendum

**DOI:** 10.3402/gha.v8.27635

**Published:** 2015-02-25

Regarding the paper titled: “The interconnected and cross-border nature of risks posed by infectious diseases” by *Jonathan E. Suk, Thomas Van Cangh, Julien Beauté, Cornelius Bartels, Svetla Tsolova, Anastasia Pharris, Massimo Ciotti, Jan C. Semenza*

Published in *Global Health Action* (section “Review Articles”) 10 Oct 2014. Citation: Glob Health Action 2014, 7: 25287 - http://dx.doi.org/10.3402/gha.v7.25287

The conflict of interest and funding statement is incorrect, and should mention that it had been developed for the HFA Thematic Review and as an input to the Global Assessment Report on Disaster Risk Reduction 2015 (GAR15).

It currently reads:

Conflict of Interest and Funding

The authors are employed by ECDC. The opinions expressed in this article do not necessarily correspond to the position of ECDC but rather of the authors.

It should read:

Conflict of Interest and Funding

The authors are employed by ECDC. The opinions expressed in this article do not necessarily correspond to the position of ECDC but rather of the authors. This paper was developed for the HFA Thematic Review and as an input to the Global Assessment Report on Disaster Risk Reduction 2015 (GAR15).

